# Supplementary material for: Content-rich biological network constructed by mining PubMed abstracts
Source: BMC Bioinformatics. 2004 Oct 8;5:147. doi: 10.1186/1471-2105-5-147 (PMC528731; doi:10.1186/1471-2105-5-147)
Supplement: Additional File 2 — The original results of the above study (non-essential files are deleted to keep the file size under the limit set by BMC bioinformatics). [file 1471-2105-5-147-S2.bz2 › chilibotAdditionalFile2/dip05/126ID9261098E470/html/RAF1_HRAS.html]

 


 **RAF1** and **HRAS** 
  
Found 26 abstracts in PubMed, retrieved 05.  
 

 What does Google say? 
 PDF only 
| .edu only 

---

**Interactive relationship** (e.g. stimulation, inhibition, etc)

**Inhibitory relationship**- Furthermore, we identified common and distinct targets in cells transformed by mutant  **HRAS** , KRAS and NRAS, as well as 61 putative target genes controlled by the RAF  [ **RAF1** ]  MEK ERK pathway in reverted cells treated with the MEK specific inhibitor PD 98059.  Ref: 10655059 Nat Genet, 2000
**Neutral relationship**- Using genomic microarray, we detected oncogene copy number gains of  **RAF1**  3p25, CTSB 8p22, PAK1 11q13, and JUNB 19p13 in 5 of 7 cases of mycosis fungoides MF Sezary syndrome SS 71%, gains of FGFR1 8p11, PTPN 20q13, and BCR 22q11 in 4 cases 57%, and gains of MYCL1 1p34, PIK3CA 3q26,  **HRAS**  11p15, MYBL2 20q13, and ZNF217 20q13 in 3 cases 43%.  Ref: 12393503 Blood, 2003
